# Empirical Bayes Analysis of Quantitative Proteomics Experiments

**DOI:** 10.1371/journal.pone.0007454

**Published:** 2009-10-14

**Authors:** Adam A. Margolin, Shao-En Ong, Monica Schenone, Robert Gould, Stuart L. Schreiber, Steven A. Carr, Todd R. Golub

**Affiliations:** 1 Cancer program, The Broad Institute of Harvard and MIT, Cambridge, Massachusetts, United States of America; 2 Proteomics platform, The Broad Institute of Harvard and MIT, Cambridge, Massachusetts, United States of America; 3 Novel therapeutics platform, The Broad Institute of Harvard and MIT, Cambridge, Massachusetts, United States of America; 4 Chemical biology program, The Broad Institute of Harvard and MIT, Cambridge, Massachusetts, United States of America; 5 Howard Hughes Medical Institute, Chevy Chase, Maryland, United States of America; 6 Dana-Farber Cancer Institute, Boston, Massachusetts, United States of America; Deutsches Krebsforschungszentrum, Germany

## Abstract

**Background:**

Advances in mass spectrometry-based proteomics have enabled the incorporation of proteomic data into systems approaches to biology. However, development of analytical methods has lagged behind. Here we describe an empirical Bayes framework for quantitative proteomics data analysis. The method provides a statistical description of each experiment, including the number of proteins that differ in abundance between 2 samples, the experiment's statistical power to detect them, and the false-positive probability of each protein.

**Methodology/Principal Findings:**

We analyzed 2 types of mass spectrometric experiments. First, we showed that the method identified the protein targets of small-molecules in affinity purification experiments with high precision. Second, we re-analyzed a mass spectrometric data set designed to identify proteins regulated by microRNAs. Our results were supported by sequence analysis of the 3′ UTR regions of predicted target genes, and we found that the previously reported conclusion that a large fraction of the proteome is regulated by microRNAs was not supported by our statistical analysis of the data.

**Conclusions/Significance:**

Our results highlight the importance of rigorous statistical analysis of proteomic data, and the method described here provides a statistical framework to robustly and reliably interpret such data.

## Introduction

Recent advances in mass spectrometry (MS)-based proteomics technology have enabled the investigation of proteomes at a systems level [1]. In particular, the ability to quantify relative protein abundance in 2 samples has made possible a plethora of proteome-wide studies including characterization of proteins or phospho-proteins that differ between 2 phenotypic states [2], measurement of changes in response to extracellular stimuli [3] or microRNA over-expression [4], [5], and analysis of sub-proteomes detected by affinity capture methods to study protein-protein interactions [6], protein phosphorylation dynamics [7]–[9], or identification of small-molecule targets [10]. Despite the immense potential and increasingly widespread application of quantitative proteomics, comparably little attention has been devoted to the analytical challenges of accurately interpreting the data and understanding the capabilities and limitations of experiments.

**Figure 1:**
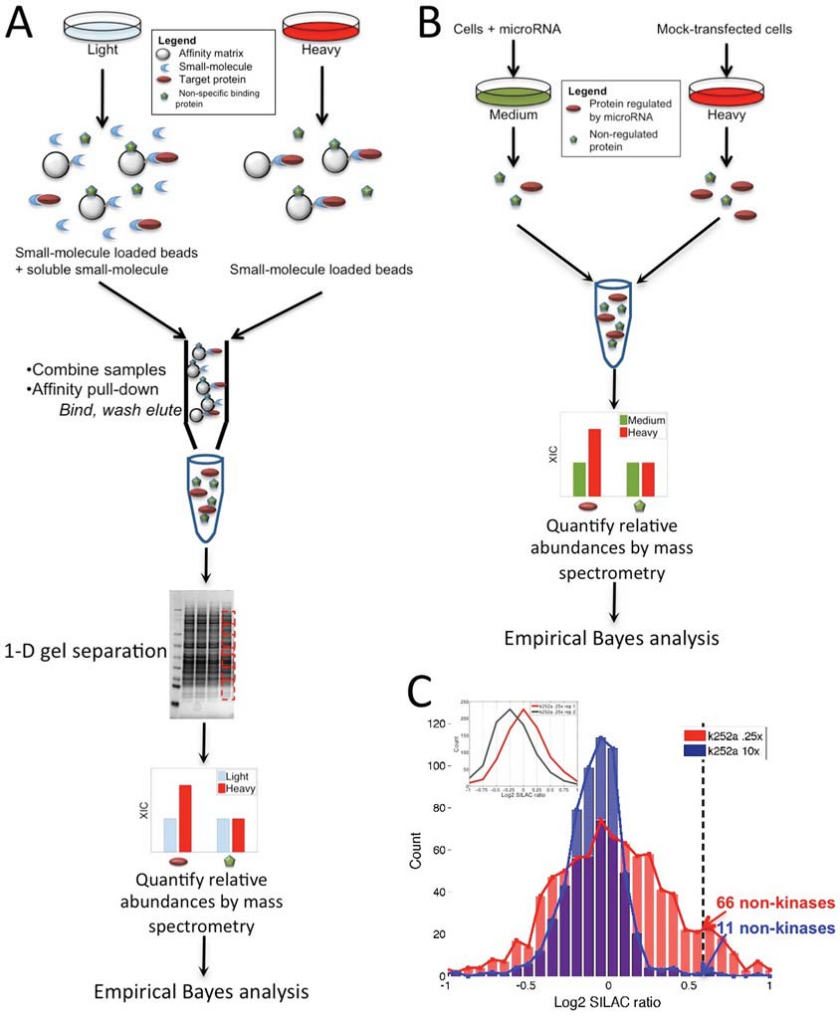
Schematics of SILAC-based experiments analyzed in our study. (A) Small-molecule target identification workflow, as described in [30]. HeLa S3 cells were cultured in “heavy” medium, containing amino acids enriched in stable isotopes (^13^C and ^15^N), and “light” medium containing forms of natural isotope abundance. Both the heavy and light lysates were incubated with small-molecule loaded beads (referred to as the affinity matrix) while the light lysate contained the addition of a soluble form of the small-molecule that competed with the affinity matrix for binding to target proteins. The red ovals represent a target protein that was bound to all 3 small-molecule loaded beads shown in this schematic for the heavy lysate, but was competed off of 2 of the 3 beads in the light lysate. The green pentagons represent a protein that bound non-specifically to the beads in both the heavy and light lysates. Proteins bound to the affinity matrix were enriched by affinity pull-down, their relative abundances were quantified by LC-MS/MS, and targets were identified by analyzing the resulting abundance ratios, 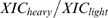, using the empirical Bayes framework described in the text. (B) microRNA workflow, as described in [4]. HeLa cells were transfected with microRNAs or mock-transfected, and pulse-labeled after 8 hours with “medium” or “heavy” amino acid isotopes. After 24 hours, samples were combined and analyzed by LC-MS/MS. The red ovals represent a protein regulated by the microRNA that was depleted in the medium lysate, and the green pentagons represent an unregulated protein of equal abundance in both lysates. (C) Two affinity pull-down experiments performed at different soluble competitor concentrations of the protein kinase inhibitor k252a displayed distinct variances of log2 SILAC ratio distributions. Applying a commonly used threshold of 1.5-fold (log2 threshold of .58) to identify significant proteins inferred 66 non-kinases (indicative of false-positives) for the high-variance experiment compared to 11 for the low-variance experiment, suggesting the necessity of experiment-specific models. (inset) The red curve represents the same k252a experiment, performed at .25x concentration, as displayed in the main plot, and the grey curve represents a replicate performed on the same cellular lysate with the same k252a concentration. This replicate experiment displayed a shift in the overall distribution due to non 1-to-1 mixing of heavy and light samples, which should be accounted for by appropriate normalization.

While several alternative approaches exist, in this work we focus on SILAC experiments [11], in which isotopically-labeled amino acids enable peptides arising from 2 different samples to be distinguishable by MS (Figure 1). A quantitative measure of differential peptide abundance is then calculated as the ratio of extracted ion intensities (XICs) between the 2 samples. A number of analytical challenges must be addressed to reliably interpret such data. In particular, analysis of mass spectra to identify peaks and map peptide sequences to proteins has been well-studied in traditional proteomics applications, and good software packages exist for generating peptide XIC ratios [12]–[14]. Moreover, several methods have been proposed for data normalization [15] and summarization of protein ratios, including averaging [13] or intensity-weighted averaging [16] of ratios for peptides identifying the same protein. However, a critical issue that remains less well-addressed is the development of statistical models to identify biologically relevant proteins based on SILAC ratio values summarized at the protein level (e.g. the median XIC ratio for all peptides identifying a protein, generally log transformed to treat over- and under-abundance symmetrically) [17], [18]. Such statistical estimates are critical since variations in relative abundance measurements arise from confounding factors such as spectral background noise, interfering signals from co-eluting peptides, differential lysis efficiencies, isotope impurities, and incomplete incorporation of the isotope label. Moreover, changes in detection instruments, experimental designs, or differential handling of samples may produce differences in technical or experimental variation (Figure 1c). Such quantitative errors must be appropriately modeled to identify ratio values attributable to true differential abundance.

Previous studies analyzing quantitative proteomics data often rely on techniques such as applying a universal fold-change threshold [7], [11], which does not account for experiment-specific differences (Figure 1c), or fitting a distribution to all ratio values [19], [20], which does not appropriately isolate only the null distribution statistics. Standard methods such as Bonferroni correction or q-value calculation have been applied to correct for multiple tests [14], [21], [22], but have been observed to be overly conservative, often computing no observations as statistically significant. Other methods require large numbers of replicates to calculate t-test p-values or to determine binding response curves at a range of soluble competitor concentrations [10]. Other methods, based on spectrum counting [23], are limited in their ability to detect low abundance proteins.

In this work, we explored the application of empirical Bayes modeling of SILAC experiments. We began by testing previously proposed methods. These include Gaussian mixture models, a type of empirical Bayes method that has been applied to quantitative proteomics data [24]–[27], as well as the approaches developed by Efron [28], [29] in the context of gene expression analysis. We found that these methods could not robustly model experimental data that contained non-Gaussian tails or regions of data sparsity, and therefore proposed a new method that more robustly fit the SILAC experimental data analyzed in our study. We then proposed multivariate statistics that integrate the results from multiple replicate experiments to compute false discovery rates, the total number of differentally abundant proteins, and statistical power.

In summary, our method models log2 SILAC protein ratio values from 1 or multiple replicate experiments and infers the full shape of class-conditional probability distributions for biologically relevant proteins (i.e. those that differ in abundance between the 2 samples) versus background. In addition to inferring the false-positive probability of each protein, the method also provides a framework to reason about inference problems not previously addressed in quantitative proteomics, such as the total number of proteins likely to be of biological relevance and the experiment's statistical power to detect them. We applied the method to detect protein targets of small-molecules based on affinity pull-down experiments and to identify differentially regulated proteins after microRNA over-expression or repression.

## Results

### Evalutation of previous empirical Bayes methods

**Figure 2:**
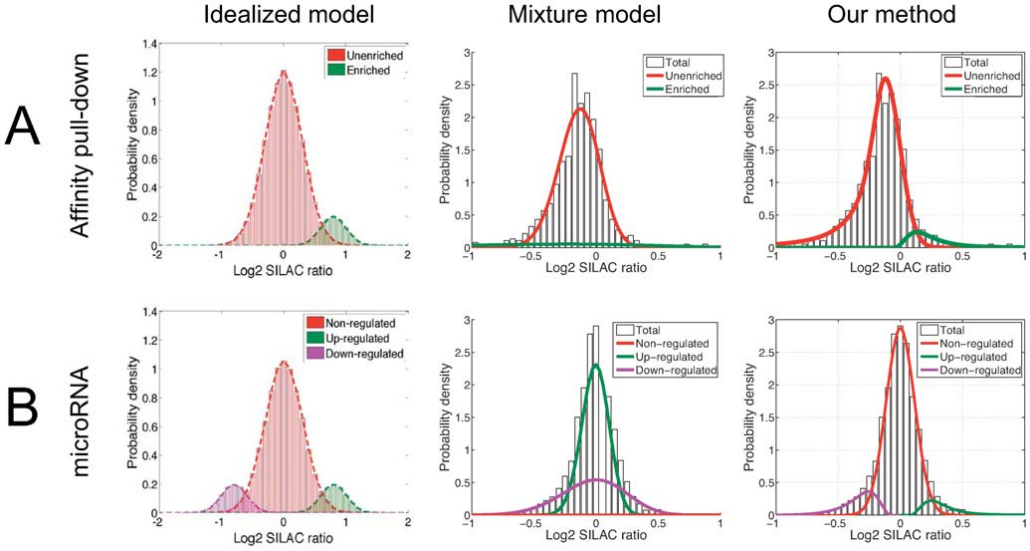
Statistical models of single-replicate experiments. For representative experiments corresponding to the 2 data modalities considered in our study (affinity pull-down and microRNA), we display the results of applying a Gaussian mixture model (following the approach of Marelli *et al.* [26]) and our empirical Bayes method. (A) Comparative models of an affinity pull-down experiment. The left panel displays an idealized representation of the desired modeling result, which distinguishes separate distributions corresponding to unenriched proteins (log2 ratio around 0) and enriched proteins (positive log2 ratios). The Gaussian mixture model failed to generate a plausible model of the experimental data (white bars) and instead represented enriched proteins using a very high-variance Gaussian (green curve) to explain the large number of data points that were not explained by the central Gaussian (red curve). Using this model, negative-ratio proteins were erroneously inferred to have a higher probability of being enriched than positive-ratio proteins. By contrast, our model correctly inferred a distribution of positive ratio values corresponding to enriched proteins, with the null distribution correctly accounting for the unenriched proteins. (B) Comparative models of a microRNA experiment. The left panel displays an idealized model that distinguishes separate distributions corresponding to proteins that are non-regulated, up-regulated, or down-regulated upon microRNA over-expression. The Gaussian mixture model failed to generate a plausible model, and instead inferred 2 distributions centered around zero, with a third high-variance distribution to explain the outliers (distributions are labeled as down-regulated, non-regulated, and up-regulated in order of their means). By contrast, our method generated a model consistent with the desired 3 distributions of down-regulated, non-regulated, and up-regulated proteins. The modeling results shown here are representative of all experiments considered in our study.

Empirical Bayes methods have been used in several quantitative proteomics studies, as well as in the related field of gene expression analysis. In particular, Gaussian mixture models are a standard and straightforward approach that has been used to model quantitative proteomics experiments [24]–[27]. However, by assuming that the data arise from a mixture of Gaussian distributions, this approach is not robust to outlier data points, and we found that such methods failed to generate plausible statistical models of the experiments considered in our study (Figure 2), which contained significantly non-Gaussian tails. We therefore motivated our approach by extending the empirical Bayes framework of Efron [28], [29], which was developed in the context of gene expression analysis and overcomes the constraints of the Gaussian mixture model by allowing more flexible modeling of the data. Briefly, this method begins by estimating the empirical marginal distribution of all data, and then estimating the portion of this distribution inferred to arise from technical and experimental variation (i.e. the null distribution). We found that proposed methods for inferring the marginal distribution, including Poisson regression models or natural splines [28], tended to over-fit the data considered in our study, which contained regions of data sparsity at the tails (Figure S1). We therefore implemented an approach that fits a flexible model to the dense central data region, but constrains the tails to be fit by a parametric model (see Methods). We then further extended the method of Efron by developing a multivariate model able to handle replicate experiments. That is, the method of Efron was developed to model a summarized test statistic (e.g. z-scores) derived from multiple experiments. For quantitative proteomics data, where each experiment measures ratio values from paired case-control samples, it is more appropriate to model each experiment separately and subsequently integrate the statistics. We therefore developed an approach in which experiment-specific models are combined to compute false discovery rates, the total number of differentially abundant proteins, and statistical power given the results of multiple experiments (see Methods).

### Application to small-molecule protein target identification

We first evaluated our methodology's ability to accurately identify the protein targets of small-molecules based on liquid chromatography tandem mass spectrometry (LC-MS/MS) relative protein abundance measurements derived from affinity pull-downs using SILAC-labeled HeLa S3 lysates (Figure 1a) [30]. We analyzed experiments using 2 different kinase inhibitor compounds and a control experiment in which ‘heavy’ and ‘light’ labeled cells were processed under identical conditions. For each experiment, the accuracy of our analytical method in recovering known targets was assessed. We note that, as with most discovery methods, gold standards are inherently imperfect as it is likely that not all target proteins of each compound have been characterized. However, we attempted to select kinase inhibitors that have been well-studied in the literature, providing as objective an evaluation framework as we could devise.

To assess the false-positive rate of our analysis procedure we first performed a control experiment, in duplicate, in which both samples were incubated with an affinity matrix loaded with the immunophilin-modulating compound AP-1497, but no soluble competitor was added. For each protein, we calculated the false-positive probability (see Methods), also called the local fdr (by convention, we write local fdr in lowercase to distinguish it from the more commonly used FDR, which is calculated based on tail areas). The local fdr calculation correctly predicted no specifically bound targets in these control experiments (all proteins were assigned a local fdr of 1), suggesting the high precision of the method. We benchmarked our method against 2 commonly used analysis strategies, not including the Gaussian mixture model approach [24]–[27], which produced uninterpretable results for our experiments (Figure 2). The first benchmark method used a universal fold-change threshold for determining significance. Two studies evaluated reproducibility using a number of analytical and experimental approaches to determine that fold-change cut-offs of 1.3 [6] or 1.5 [7] in either replicate experiment reliably eliminated analytical errors. We used the 1.5 ratio cut-off, which produced better results for our experiments. This approach identified 5 proteins passing the fold-change criteria, suggesting that such methods likely incur false-positives. The second benchmark method used a Gaussian error model to determine significance [19], [20]. We note that several studies have used Bonferroni correction and other conservative approaches to correct for multiple hypotheses, and we observed that such approaches often eliminated all proteins as being non-significant, consistent with previously reported findings [21]. To avoid such overly-conservative corrections and to enable direct comparison with our method, we computed false-positive probabilities for each log2 SILAC ratio based on q-values [31] computed from the Gaussian error model (see Methods). The Gaussian method also correctly predicted zero significant targets in the control experiments.

**Figure 3:**
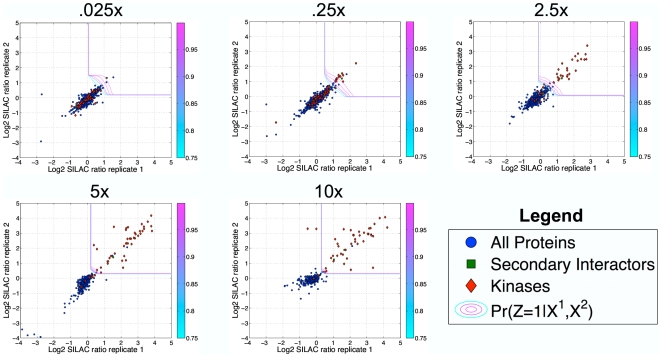
Empirical Bayes model applied to replicate k252a experiments. Scatter plots display log2 SILAC ratio values for replicate experiments performed using k252a. Plots are arranged in order of increasing soluble competitor concentration. Contour lines display the predicted probability of binding k252, 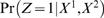, ranging from 99.9% to 75% from outermost to innermost. Red diamonds represent protein kinases, green squares represent proteins known to exist in complex with a protein kinase that was identified as a k252a target, and blue circles represent all other proteins.

We next tested our ability to identify the protein targets for broad-specificity compounds. We performed replicate experiments at soluble competitor concentrations of 0.025x, 0.25x, 2.5x, 5x, and 10x of k252a, a staurosporine analog that binds broadly to protein kinases. In this experimental design, the soluble competitor should compete proteins specifically bound by k252a off of the affinity matrix in the light sample, but non-specific binders should remain, causing k252a target proteins to be distinguishable by high SILAC ratios (Figure 1a). We evaluated our predictions with respect to known human protein kinases [32]. This analysis indicated that our method achieved high precision in discriminating kinases from non-kinases (Figure 3 and Table 1). For each of the 4 experiments performed at higher soluble competitor concentrations, over 86% of the inferred targets (local fdr cut-off .01) were protein kinases, with up to 41 kinases inferred as targets (Table 1). In addition, the validity of multiple non-protein-kinases inferred as targets was supported by additional evidence. For example, 4 non-kinases were identified as targets in the 10x experiment: TPRKB and CCNB1 form complexes with the protein kinases TP53RK and CDC2, respectively, both of which were identified as k252a targets; a third non-kinase inferred target, the oxidoreductase NQO2, has recently been demonstrated to bind specifically to the kinase inhibitor imatinib [10]; and the fourth non-kinase target, OSGEP, is a novel prediction, and was identified at both the 5x and 10x soluble competitor concentrations, suggesting that this was a reproducible finding. The complete list of predicted targets for each experiment using each analysis method is given in Table S1.

**Table 1:** Evaluation of predicted small-molecule targets.

Small-molecule	Concentration	Total detected	Known targets detected	Total significant	Known targets significant	Precision	Recall
k252a	.025x	584	42	3	0	0%	0%
k252a	.25x	697	55	15	13	87%	24%
k252a	2.5x	530	36	29	25	86%	69%
k252a	5x	637	47	46	41	89%	87%
k252a	10x	540	43	43	39	91%	91%
SB-202190	10x/100x	276	6	10	6	60%	100%
AP-1497	0x	514	0	0	0	N/A	N/A

For each experiment we evaluated the list of targets inferred by our model compared to a list of known targets, defined for k252a as human protein kinases [32] and for SB-202190 as those identified by Karaman *et al.* [33]. “Detected” refers to all proteins detected by LC-MS/MS and “significant” refers to those inferred as significant by our model (local fdr threshold .01). Precision was calculated as the percent of proteins inferred as significant by our model that were also known targets, and recall was calculated as the percent of known targets detected by LC-MS/MS that were also inferred as significant by our model. Because our lists of known targets were incomplete, precision statistics were likely underestimated. Targets with additional evidence of binding each small-molecule are annotated in Table S1.

**Figure 4:**
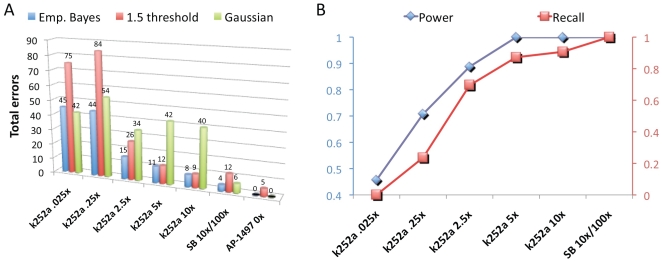
Evaluation of methods applied to affinity pull-down experiments. (A) For each experiment we calculated the total number of errors (false-positives plus false-negatives) based on each analysis method, defining known targets for k252a as human protein kinases [32] and for SB-202190 as targets identified by Karaman *et al.* [33]. Zero targets should be identified for the control experiment (AP-1497, no soluble competitor), thus the total errors are the number of identified proteins. These comparisons consistently illustrated the superior performance of our method. (B) Increased statistical power correlated with the percent of known targets identified as significant by our model (recall).

In comparison, the Gaussian model identified only the highest confidence interactions, yielding a maximum of 5 predicted targets in any of the experiments (cut-off .01). We note that even relaxing the cut-off to .2 would produce only 12 and 21 predicted targets for the 5x and 10x experiments, respectively. Interestingly, although the proteins identified as significant by our method for the 5x and 10x experiments were largely in agreement with the 1.5 ratio threshold determined by Blagoev *et al.* [7], the 1.5 threshold yielded dramatically more false-positives for the 3 experiments at lower concentrations (Figure 4a and Table S1). Empirical Bayes models of the 2 higher concentration experiments predicted sharp discriminatory boundaries near a ratio of 1.5; however, the 3 lower concentration experiments, which were performed on a different set of lysates from a separate culture of cells, displayed more experimental variability (Figure 1c), and our model predicted a corresponding increase in the significance thresholds (Figure 3). Indeed, the majority of proteins identified in the 3 lower concentration experiments using the 1.5 ratio cut-off, but not significant by our model, showed no evidence of being associated with kinase biology, supporting the use of models such as ours to capture experiment-specific variability.

Having demonstrated that the local fdr statistic reliably produced a low false-positive rate for the k252a experiments, we then evaluated whether statistical power diagnostics could be used to reason about the expected false-negative rate. Our analysis predicted that statistical power increased with increasing k252a soluble competitor concentration, and, consistent with this prediction, the percent of kinases detected by LC-MS/MS that were statistically significant (recall) also increased (Figure 4b). For the 5x and 10x experiments, we predicted near 100% statistical power, in agreement with our gold standard comparison (Figure 3), which showed that protein kinases (red diamonds) were well-separated from non-protein-kinases (blue dots). Statistical power decreased with decreasing soluble competitor concentration, indicating that the SILAC ratio sub-distribution derived from target proteins merged with the sub-distribution derived from non-specific binding proteins, consistent with the observation that SILAC ratios of protein kinases began to overlap with those of non-protein kinases (Figure 3).

**Figure 5:**
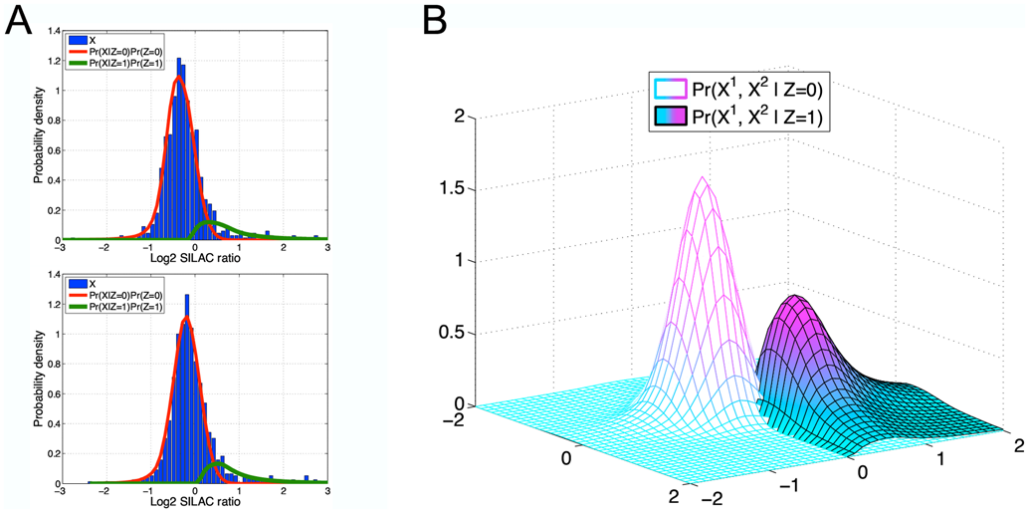
Increased statistical power from replicate experiments. Modeling of class-conditional probability distributions allows statistical reasoning about the power of an experiment. (A) Inferred distributions from individual replicate experiments of k252a at 2.5x concentration showed that the distribution arising from target proteins (green curve), Pr(X|Z = 1)Pr(Z = 1), had significant overlap with the inferred null distribution (red curve), Pr(X|Z = 0)Pr(Z = 0), corresponding to power statistics of 75.6% and 83.4%, respectively for the top and bottom plots. (B) Theoretical modeling of the inferred bivariate class-conditional probability distributions from replicate experiments showed that the distribution arising from target proteins (solid curve), 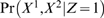, was well-separated from the inferred null distribution (mesh curve), 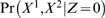, corresponding to an increased power statistic of 91.5%. For ease of visualization, bivariate class-conditional probability distributions are not scaled by their prior probabilities.

Interestingly, our model inferred roughly the same total number of targets in the 2.5x experiment as for the higher concentrations (Table S1), but predicted that the 2.5x experiment had reduced power to reliably detect them, correctly describing the fact that roughly the same total number of kinases were detected by LC-MS/MS at each of these concentrations, but many of these kinases could not be robustly separated from the background at 2.5x concentration. At the very low concentrations (.25x and .025x) both the total number of inferred targets and statistical power were reduced, as the SILAC ratios for some kinases showed no enrichment towards positive values (Figure 3). Our model also predicted that, for all experiments considered here, substantial statistical power was gained by performing replicate experiments, as the bivariate class-conditional probability distributions of SILAC ratios from targets versus non-targets were more robustly separated than those from single experiments (Figure 5).

Overall, these results suggested that the experimental methodology, and associated statistical model, provided high precision in identifying small-molecule targets with a low false-positive rate. Increasing the soluble competitor concentration led to identification of more true targets without an associated increase in false-positives. These conclusions were supported by comparison with our list of protein kinases, but we stress that, absent a gold standard list, the empirical Bayes methodology and power diagnostic accurately described these features of the experiments from statistical modeling of the data alone.

We next examined our ability to correctly detect targets of a more specific small-molecule. We tested SB-202190, a kinase inhibitor of MAPK14 (also known as p38). This compound was recently evaluated in an *in vitro* competition assay against a panel of 287 distinct human protein kinases, representing ∼55% of the predicted human protein kinome [33], and the authors detected 39 targets of SB-202190 with K_D_<10 µM. Based on our conclusions from the k252a analysis, we performed single SILAC experiments using soluble competitor concentrations of 10x and 100x and applied our statistical model to assess the probability that each detectable protein was bound by SB-202190 given these 2 SILAC ratios. Only 6 of the 39 targets reported by Karaman *et al*. were detectable by LC-MS/MS in our pull-down experiments (including MAPK14), and all 6 of these proteins were significant using the local fdr cut-off of .01 (Table 1). Four additional targets – TGFBR1, CSNK1A1, CHD6 and FAM83G – were also identified at this threshold. TGFBR1 was tested in the study of Karaman *et al*. but not reported as bound by SB-202190. However, it was reported as a low affinity (7.1 µM) binder of the closely related and structurally similar SB-431542, which showed an extremely similar binding profile to SB-202190 in this experiment. It is therefore likely that TGFBR1 also binds with low affinity to SB-202190 but was not detected at the threshold of 10 µM used in this study. CSNK1A1 was not tested by Karaman *et al*., but 3 related proteins – CSNK1A1L, CSNK1D, and CSNK1E – were tested and all found to bind SB-202190. CHD6 and FAM83G are not kinases and therefore were not tested by Karaman *et al*., although CHD6 is known to bind ATP [34] suggesting that it may also bind kinase inhibitors, which interact with ATP-binding domains of protein kinases. The calculated power statistic for this experiment was >99%, indicating that the inferred sub-distribution arising from target proteins was completely separated from the inferred null distribution, supporting our conclusion from the k252a experiments at higher soluble competitor concentrations, and in agreement with the statistical significance of all proteins identified by Karaman *et al.* and also detected by LC-MS/MS. In comparison, the Gaussian model eliminated all targets as non-significant, while the fold-change method inferred the same 10 targets as our method, but also inferred an additional 8 targets, none of which have evidence of binding SB-202190 (Figure 4a and Table S1).

### Application to microRNA experiments

Having demonstrated our method's utility for small-molecule protein target identification experiments, we sought to evaluate its application to an expanded range of quantitative proteomics experiments. We therefore applied the statistical method to the analysis of a recent study by Selbach *et al.* [4] that used pSILAC (a variant of SILAC used to measure only newly synthesized proteins, rather than total protein abundance) to identify proteins differentially regulated upon microRNA over-expression or repression (Figure 1b).

To evaluate whether proteins differentially regulated by microRNA over-expression or repression could be accurately identified using our empirical Bayes strategy, we applied our model to assign a probability that each protein is differentially regulated in the 6 experiments performed by Selbach *et al*. These include over-expression of human microRNAs miR-1, miR-155, let-7b, miR-16, and miR-30a and knockdown of let-7b, performed in single replicate. Analogous to the small-molecule target identification application, we assumed a 3-class model of SILAC ratio values, in this case representing proteins up-regulated, down-regulated, and not differentially regulated upon microRNA over-expression or repression. In contrast to the conclusion of Selbach *et al*. that each of the 5 tested microRNAs regulated most of the 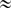
3,000 proteins detected in the corresponding experiment, our analysis indicated that only between 5 and 13 percent of proteins detected in each experiment were down-regulated by microRNA over-expression (or up-regulated by knockdown). The statistical power for each experiment ranged from 63.8% to 76.5%, indicating that the full range of differentially regulated proteins could not reliably be detected (Table S2). We stress that even for underpowered experiments, the total number of differentially expressed proteins can still be inferred (see Eqn. **(4** **)** in Methods).

We evaluated the accuracy of our predictions by considering microRNA seed sequence enrichments in the 3′ UTRs of mRNAs corresponding to predicted differentially regulated proteins. Directly relating the accuracy of predicted targets to seed sequence enrichments is challenging, because the presence of a seed sequence is not directly predictive of microRNA regulation, and differentially regulated proteins may contain a mixture of those directly targeted by the microRNA as well as secondary effects. In effect, the presence of a seed sequence is a noisy indicator of differential expression upon microRNA over-expression or repression. However, we may evaluate our predictions with respect to this noisy indicator by using the strategy employed by Margolin *et al.* [35]. That is, we may evaluate the enrichment of seed sequences as a function of the local fdr threshold, compared to a set of proteins unlikely to be regulated by the microRNA, providing a standardized benchmark quantity for all experiments, irrespective of background site enrichment. We expected that proteins inferred as non-significant by our model would show no enrichment in the corresponding microRNA seed sequence while those identified with high-confidence as being differentially regulated would be enriched in seed sequences, even if some indirectly regulated proteins were included as well.

**Figure 6:**
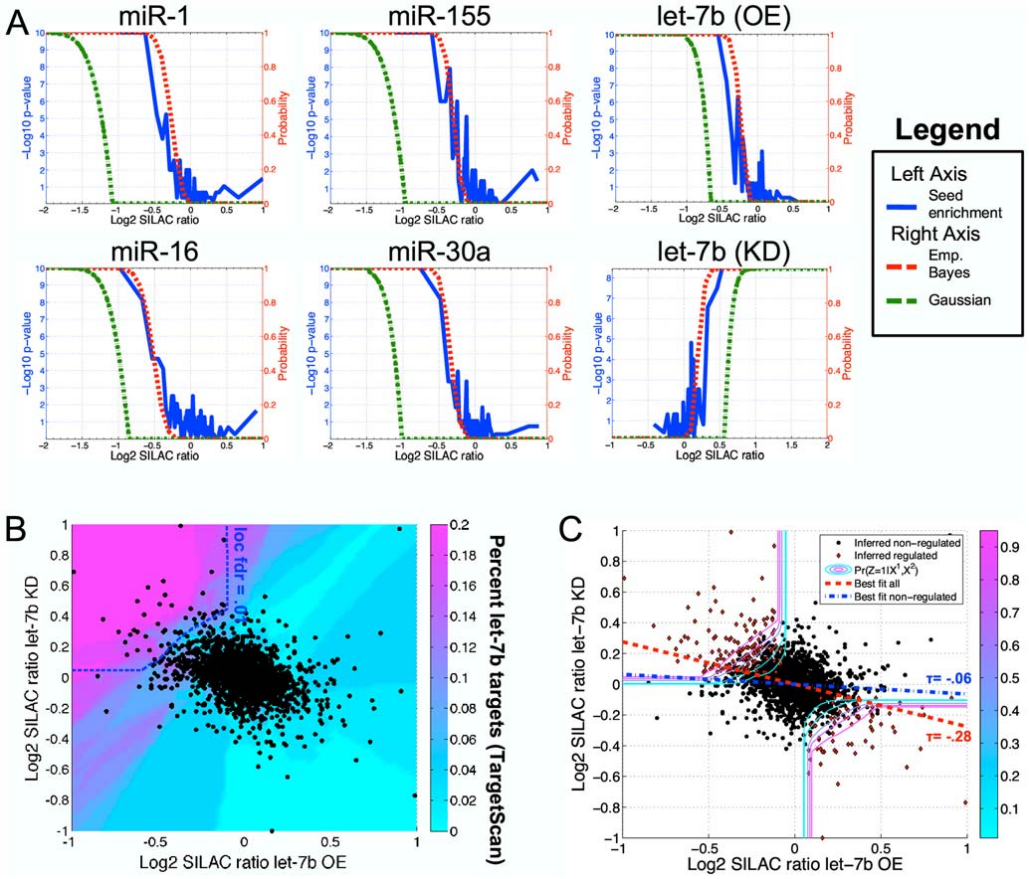
Empirical Bayes analysis of microRNA experiments. (A) For single replicate models, microRNA seed sequence enrichments (blue curve) corresponded with inferred probabilities of differential regulation computed by the empirical Bayes method (red curve), while Gaussian error modeling (green curve) eliminated the majority of regulated proteins. Sequence analysis was performed using bins of fifty proteins (plotted at the mid-point of each bin) sorted by ascending SILAC ratios, and computing the *p*-value of microRNA seed sequence (positions 2–8) enrichment, based on the hypergeometric distribution, against a background of the one-third of proteins with the largest SILAC ratios (or lowest ratios for the let-7b knockdown experiment). Similar results were obtained using different bin and background sizes, as well as different seed sequence definitions or algorithmically predicted targets (Figure S5). (B) Log2 SILAC ratios from the let-7b over-expression experiment were plotted against log2 SILAC ratios from the let-7b knockdown experiment. Colors indicate the percent of predicted targets, using the Target Scan algorithm [36], for each pair of ratio values, averaged over the 100 nearest neighbors by Euclidean distance. Only proteins inferred as significantly down-regulated upon let-7b over-expression and up-regulated upon let-7b knockdown (dotted blue curve) appeared enriched in let-7b target sites. (C) Data points display the same scatter plot as in (B), with contour lines representing inferred probabilities of differential regulation (assuming significant proteins move in opposite directions upon let-7b over-expression or knockdown). For each of the 225 regions in an evenly spaced grid over the plot's domain, we randomly selected a percent of proteins according to the inferred probability of differential regulation (red points). These proteins are representative of those inferred to be regulated by let-7b. The overall data displayed a large negative correlation (τ = −.28), but the correlation based only on inferred non-regulated proteins was reduced to τ = −.06. We used Kendall's τ rank correlation coefficient to provide robustness against potential outlier points that would be eliminated only from the upper-left and lower-right quadrants of the plot, and therefore overstate our finding by reducing Pearson correlation to near zero.

Our analysis indicated that, in general, empirical Bayes predictions were in quantitative agreement with seed sequence enrichment analysis, thus validating our findings (Figure 6a). Proteins with ratio values predicted by our model to represent experimental variation rather than true differential regulation (i.e. those with near zero probability of significance), showed no enrichment in microRNA seed sequences. As the predicted probability of differential regulation increased, we observed a corresponding increase in the enrichment of seed sequences, with all proteins in the high-confidence range being highly enriched. In contrast, the Gaussian method and the fold-change method both inferred as non-significant the majority target proteins. We estimated that the 1.5 ratio cut-off eliminated at least two-thirds of truly differentially regulated proteins in 5 of the 6 experiments (Table S2). To obtain a larger list of putative targets, Selbach *et al*. compiled a list of proteins with log2 SILAC ratios less than −.1 (or greater than .1 for the knockdown experiment). Our analysis predicted that more than half of all proteins identified using this threshold were likely to be false-positives (Table S2). Supporting this observation, the 200 proteins with the largest log2 SILAC ratios less than −.1 (or smallest ratios greater than .1 for the knockdown experiment) were not statistically significantly enriched in microRNA seed sequences (*P*>.05 for all experiments).

Interestingly, the authors of this study observed an overall negative slope of ratio values in the let-7b over-expression versus repression experiments, suggesting that let-7b may globally regulate the production of most of the 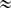
3,000 proteins detected in the experiments, in contrast to our prediction that only several hundred proteins are regulated in each experiment. We therefore applied our model to assess the probability that each protein is up-regulated upon let-7b knockdown and down-regulated upon let-7b over-expression (or down-regulated upon knockdown and up-regulated upon over-expression). Consistent with our observation from the affinity pull-down experiments, when considering both the over-expression and knockdown experiments together, statistical power was increased to 88.4% (compared to 68.1% and 67.1% for each experiment considered individually). Moreover, in agreement with our model, we observed that only proteins in the statistically significant region were enriched in let-7b targets predicted by the TargetScan algorithm [36](**Figure 6b**). While the remaining proteins may indeed be regulated by non-seed mediated mechanisms, we suggest an alternate possibility that a limited number of differentially regulated proteins (including those up-regulated upon over-expression and down-regulated upon knockdown) may display ratio values that overlap with those of non-regulated proteins, influencing the overall slope of the scatter plot. Consistent with this hypothesis, computing the slope based only on inferred non-regulated proteins (by eliminating data points in proportion to their inferred probability of regulation) reduced Kendall's τ correlation coefficient from .28 to .06 (**Figure 6c**). Although suggestive, we recognize that sequence enrichment analysis does not constitute proof of our hypothesis, and specific follow-up experiments are necessary to validate the full range of let-7b targets. However, our results, taken together, indicate that the data may not support the contention that let-7b globally regulates protein production, as previously suggested [4].

## Discussion

Modern high-throughput technologies in experimental biology produce large-scale data sets consisting of hundreds or thousands of measurements, presenting simultaneous inference challenges not anticipated by classical statistical methods that were designed for problems with small numbers of data points and limited computational power. Commensurate advances in statistical inference methods are required to maximally exploit the information generated by this technological revolution. The past decade has thus seen a flourishing of novel statistical and computational methodologies (and resurrection of underdeveloped methodologies) designed to tackle large-scale simultaneous inference tasks, as epitomized by gene expression micorarrays, but extending to many other high-throughput technologies. Quantitative proteomics methods are only recently becoming viable in large-scale and can benefit from standardized analysis methods built from the advances made in related statistical inference problems.

Although empirical Bayes methods have been explored in proteomics analysis, and have been well-studied in related fields such as gene expression analysis, several modifications improved their ability to model the SILAC experiments considered in our study. In particular, the Gaussian mixture model approaches that were previously used in proteomics applications were not robust to experiments with non-Gaussian tails, whereas the density estimation methods developed for gene expression analysis tended to over-fit regions of data sparsity. We therefore modified the method proposed by Efron to obtain more robust models of the experimental data analyzed in this paper. We further extended the method of Efron to facilitate its application to quantitative proteomics experiments by developing multivariate statistics that integrate the results of experiment-specific models of multiple replicate experiments to compute false discovery rates, statistical power, and the total number of differentially abundant proteins.

Overall, we believe that empirical Bayes methods, and the particular novel aspects described in this work, will be a powerful addition to the analytical approaches to quantitative proteomics experiments. Empirical Bayes methods are designed to leverage aspects of both Bayesian and frequentist statistical inference by using the powerful and flexible reasoning tools of Bayesian statistics, but exploiting the massively parallel structure of the data to infer prior probability distributions in a frequentist-type setting. This data-driven approach relies on minimal assumptions and enables transparent and consistent inference of class-conditional probability distributions, using a highly flexible model of the marginal and a Gaussian model of the null distribution. While the null distribution model may produce some inaccuracies for data with significantly non-Gaussian tails, we note that the 2-class model is unidentifiable without restrictions on the form of this distribution [37], and Gaussian distributions are widely used in error modeling. For the experiments considered here our analysis indicated that use of a Gaussian model did not produce significant errors, as zero proteins were significant in the control experiment with no soluble competitor, and the models of the other affinity pull-down experiments appeared to yield very low false-positive rates.

The Bayesian construction enables a principled framework that encompasses a number of inference tasks about the data and can be used both to predict significant observations (e.g. small-molecule targets) and reason about the capabilities and limitations of an experiment (e.g. statistical power). We believe these tools can be used to guide experimental design and inform follow-up experiments in a cost-effective manner. We have demonstrated the use of power analysis of various affinity pull-down experiments to suggest high soluble competitor concentration and replicate experiments as the optimal experimental design, predicted to yield near 100% statistical power. Consistent with this prediction, our models of the high-concentration replicate experiments for both k252a and SB-202190 appeared to yield near optimal discrimination of known targets versus non-targets that were detected by LC-MS/MS. The prediction that nearly all detected targets could be reliably identified as significant by our model indicated that the dominant cause of false-negatives was likely proteins not detected by LC-MS/MS. This analysis suggests that further experimental improvements should focus on increasing the number of detected proteins, for example by increasing the amount of input protein, the amount of small-molecule loaded onto the affinity matrix, or the sample fractionation.

Our statistical descriptions of the microRNA experiments were different from those of the affinity pull-down experiments. We predicted that several hundred proteins were differentially regulated in each microRNA experiment. However, because the magnitude of regulation was subtle, not all differentially regulated proteins could be reliably separated from the experimental variation, yielding sub-optimal power diagnostics. We believe that such a precise description of an experiment has useful implications for understanding the underlying biology. For example, our observation that some microRNA targets were only subtly regulated is significant considering recent evidence that such subtle regulatory changes can have large phenotypic effects [38]. This suggests that the full range of identified differentially regulated proteins are important for follow-up investigation; however, the underpowered nature of the microRNA experiments indicated that all regulated proteins could not be identified without encountering a large number of false-positives (as can be predicted by the local fdr statistic). The Bayesian inference framework enables natural incorporation of additional information, which we suggest may be combined with the weak evidence provided by underpowered experiments to increase the overall statistical power. We demonstrated a simple example of increasing statistical power by incorporating replicate experiments under the conditional independence assumption, but additional data sources, as well as prior information, may also be naturally integrated with our methodology. For example, we suggest that our models of the microRNA proteomics experiments may be integrated with probabilistic models that predict microRNA targets based on sequence analysis [39], into a unified probabilistic inference framework that may achieve increased statistical power to reliably discriminate the full range of microRNA targets. The power diagnostic therefore provides a useful indicator of when incorporation of additional information may increase the utility of predictions.

Although assessing the performance of an analytical method is challenging when true gold standards are not known, several criteria indicated the robust performance of our method for both data types considered in our study. For the affinity pull-down experiments our method correctly recovered kinase targets of k252a, previously reported targets of SB-202190, and no targets in the absence of soluble competitor. For the microRNA experiments, our results were in agreement with seed-sequence enrichment analysis. Finally, for both types of experiments, our method produced more accurate models of the experimental data than existing methods.

Although evaluation of our method's general applicability will require further studies, we believe that the minimal assumptions and demonstrated robustness of our approach suggest that it should generalize to other SILAC data sets similar to those analyzed in our study. The strategy employed for the small-molecule target identification experiments is similar to that of several other applications that analyze affinity-purified sub-proteomes from SILAC-labeled lysates, including to study protein-protein interactions using recombinant protein baits [6]; protein-peptide interactions using synthetic peptide baits [12]; global kinome phosphoproteomics using kinase-selective baits [8]; or phosphotyrosine-dependent signalling using anti-phosphotyrosine antibody baits [7], [9]. Moreover, the results obtained from analysis of the microRNA experiments suggest that the method should generalize to other experiments analyzing SILAC-labeled lysates to identify proteins or phosphoproteins that differ in abundance between 2 conditions, for example to compare different phenotypes [2], [24], [40]; primary cells versus cell lines [41]; or response to perturbation [3], [42]. Although we only considered SILAC-based experiments in this study, in principle the method should also apply to analysis of experiments performed using other stable isotope labeling strategies such as chemical modification-based approaches using ICAT [43] or iTRAQ [44].

We believe that the analytical methodology developed here will be a useful addition to the wealth of data that can be generated by modern quantitative proteomics methods. In particular, the coupling of quantitative proteomics with an appropriate statistical framework appears to now make feasible the routine identification of protein targets of small-molecules. This capability has the potential to greatly enhance cell-based chemical biology screens, where the protein targets of active compounds have often remained unknown. More broadly, the availability of a generalizable, statistically principled analytical framework for proteomic analysis should facilitate the incorporation of proteomic data into systems biology studies.

## Methods

### Statistical model

We describe our analytical method in the context of the affinity pull-down experiments in SILAC labeled lysates (Figure 1a), but it is applicable to any quantitative proteomics data set. The method takes as input log2 SILAC protein ratio values, corresponding to relative abundances, for 1 or multiple replicate experiments. In our affinity pull-down data set we observed minimal increase in the variance of ratio values at low intensities (Figure S2), and thus summarize data at the protein level using the common strategy of calculating the median log2 XIC ratio value across peptides identifying the same protein [2], requiring at least 2 unique peptide hits in any replicate experiment for confident protein identification.

We formulate the inference task as one of binary classification between small-molecule targets and non-targets (we assume non-targets have low enough affinity for the soluble competitor to have negligible effect on the SILAC ratio). We thus seek to compute the Bayes posterior probability that a protein is not bound by the test compound given the measured SILAC ratios from 1 or multiple experiments. For a single experiment, this posterior probability is called the local fdr [45]:
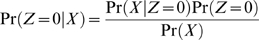
(1)where *Z* is a binary variable taking the value of zero if the protein is not bound by the compound, and *X* is a measured log2 SILAC ratio. In this 2-class model, the probability that a protein is bound by the test compound given the SILAC ratio is simply calculated as 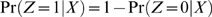. In the empirical Bayes framework, the local probability models in Eqn. (1) are estimated from the data, under the assumption of exchangeability. That is, that all proteins have the same prior probabilities of being bound or unbound.

Pr(X), the denominator of Eqn. (1), defined as the marginal distribution of all *X*'s, is estimated using all of the data in the experiment. Approaches for estimating this distribution, proposed in the context of microarray analysis, include fitting maximum likelihood estimates of high-order polynomials [28] or mixture models [46], [47]. However, it is difficult to apply these methods to the data considered here since very few proteins may be bound by the compound, yielding sparse data at the tails of the histogram, which tend to be over-fit by models that allow too much flexibility (Figure S1). By contrast, data arising from the unbound proteins are tightly centered around the mixing ratio (log2 ratio close to zero), and can be estimated accurately using flexible parametric or non-parametric methods. We therefore infer the central part of the distribution using a Gaussian kernel estimator, and fit the tails with generalized Pareto distributions. Although other heavy-tail distributions could reasonably be applied as well, generalized Pareto distributions were developed based on theoretical arguments to accurately model the tails of a large number of distributions [48], and we observed that this approach could robustly model the different experiments considered in our study. In this work, we fit the Pareto tails using log2 ratios 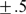 from the mode of the data, corresponding to the region where data was sparse. Although this choice is arbitrary, we found that variations in this parameter had negligible effect on the inference results, as the method was robust over a large range of parameter choices (Figure S3). After calculation of the central distribution and Pareto tails, we then smooth the resulting piecewise distribution using a cubic spline to infer a functional estimate of the complete marginal, Pr(*X*).

**Figure 7:**
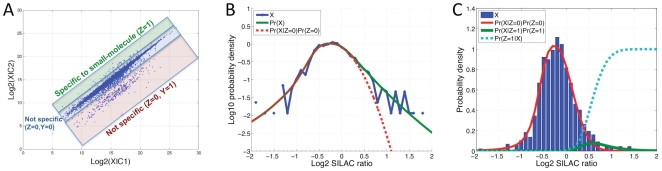
Schematic of empirical Bayes model for single replicate experiments. (A) Example scatter plot of log2 peptide XIC1 versus XIC2 values with colored boxes schematically representing our formulated 3-class model. The green box represents peptides corresponding to small-molecule-specific target proteins. The majority of ratios (blue box) were clustered along the diagonal of the plot and represent peptides corresponding to proteins that were pulled down in the experiment but did not bind specifically to the small-molecule. A third group of peptides (red box) had negative log2 ratio values and were visually separated from the cluster of peptides along the diagonal. These negative ratios were likely caused by proteins that precipitated out in the light sample due to excess concentration of the soluble competitor small-molecule. (B) The blue curve represents a histogram of protein ratio values for a k252a experiment performed at .25x concentration (y-axis in log scale, histogram bins with zero counts are shown as missing data points). The green curve represents the inferred marginal distribution of all data, Pr(X), and the red curve represents the inferred sub-distribution corresponding to non-targets, Pr(X|Z = 0)Pr(Z = 0). (C) Using data from the same experiment as in (B), the posterior probability, Pr(Z = 1|X), was plotted as the dotted cyan curve (y-axis linear scale). The blue bars represent a histogram of the data. The red curve represents the inferred sub-distribution corresponding to non-targets, Pr(X|Z = 0)Pr(Z = 0), and the green curve represents the inferred sub-distribution corresponding to targets, Pr(X|Z = 1)Pr(Z = 1).

The numerator of Eqn. (1), 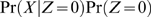, represents the contribution to the marginal distribution arising from the unbound proteins. Before describing estimation of this quantity, we consider 2 differences between the data presented here and the data used in empirical Bayes analysis of microarray experiments. First, in the small-molecule target identification application, values of interest only manifest as positive log2 ratios, requiring 1-tailed significance tests. Second, values for unbound proteins may be affected by several different processes. The majority of unbound proteins have log2 ratios near zero, with deviations caused by technical and experimental variation. There is often an additional class of proteins, with distributions clearly separated from the first class, that have negative log2 ratio values (Figure 7a, red box). While the cause of these negative values is not fully known, they may be due to proteins that precipitate out once the soluble competitor concentration becomes too high. This hypothesis is supported by our observation that these negative values correspond to visual precipitation in the sample with soluble competitor added. We note that they are not generally due to mass spectrometry carryover and contaminants as they localize to specific bands in our GeLCMS analysis, indicating that these proteins were resolved in SDS-PAGE. Furthermore, differential abundances of corresponding molecular weight proteins were also observed in unmixed gel visualizations of the same pull-down samples. Therefore, although we retain the form of Eqn. (1) for consistency with the local fdr literature, in this application we hypothesize a 3-class model (shown schematically in Figure 7a) in which the numerator of Eqn. (1) is separated into 2 components:
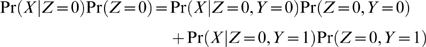
where *Y* is a binary variable taking a value of zero if the corresponding protein arises from the component of the null distribution representing technical and experimental variation, and a value of 1 if it arises from the component of the null distribution with negative log2 ratio values not explained by technical and experimental variation. We note that the same 3-class model also applies to experiments designed to detect both significant positive and negative ratio values, for example, the microRNA experiments in which the 3 classes represent proteins up-regulated, down-regulated and not differentially regulated. In this application, down-regulated proteins would correspond to the class (*Z* = 0, *Y* = 1).

We next estimate each of these components. The term 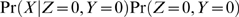 corresponds to the null distribution typically inferred in microarray local fdr applications and can be estimated by making the “zero assumption” that the data around the central peak of the histogram arises mostly from proteins in this class (i.e. *Z* = 0, *Y* = 0). Assuming a Gaussian distribution for the log2 ratio values of this class, we fit a quadratic function to the central region of 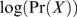 [49]. Based on previous estimates of experimental variability [50], in this work we defined this central region as log2 ratios 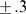 from the mode of the data, and because the central region of the data was well fit by a Gaussian (Figure 7b), we found that the fit was robust to the amount of data used (Figure S3). For the microRNA experiments, the null distribution was calculated using the central 50% of the data (mean window size .22), and results were again robust over a large range for this parameter (Figure S3). By construction, the data around the central peak is assumed to arise from. Therefore, the other 2 sub-distributions, 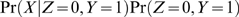 and 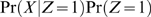 are assumed to be completely separate and arise, respectively, from data on the left and right side of the central peak. Because the 3 sub-distributions are mutually exclusive and exhaustive:
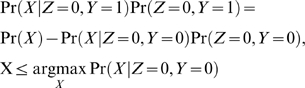
(2)and
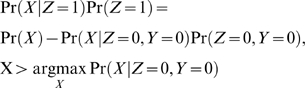
(3)and are set to zero for values of X not specified in the arguments of Eqns. 2 and 3. The proportion of unbound proteins can then be inferred by integration of the numerator of Eqn. (1):
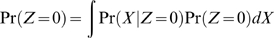
(4)and the proportion of bound proteins is calculate as 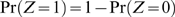.

For replicate experiments we assume that the data are conditionally independent given the class, *Z*, and compute the Bayes posterior probability that a protein is unbound by the test compound given *M* SILAC measurements as:
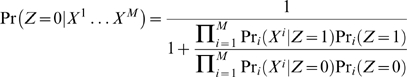
(5)where 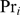 represents the probability model inferred in the i^th^ experiment. The probably that a protein is bound by the test compound given *M* SILAC measurements is calculated as 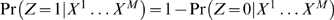.

#### Power diagnostics

The probability distributions inferred in the previous section can be used to reason about the statistical *power* of an experiment, defined as the probability that the experiment will not make a Type II error (i.e. reject a protein that is truly bound by the small-molecule). In Bayesian terms, a measure of statistical power is 1 minus the expected local fdr under the inferred class conditional probability distribution given that the protein is a target [49]. In our application we compute this measure of statistical power as:
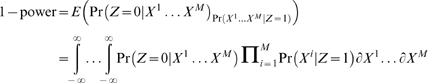
(6)


### Gaussian Error Model

To enable direct comparison between our local fdr values and Benjamini Hochberg tail-area FDR values [51] calculated based on standard Gaussian error modeling, we transform the tail-area FDR values to probability estimates at each data point using the procedure described here. Although derived in a frequentist context, the Benjamini Hochberg FDR can also be written in Bayesian form [52]:
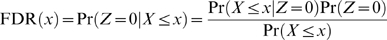
(7)


The term 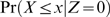 is the familiar *p*-value, which we estimate using a Gaussian error model, and 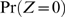 is set to 1. The Benjamini Hochberg procedure then estimates the denominator of Eqn. **(7)** using the empirical CDF, 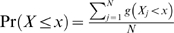, where 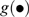 returns a value of 1 if the argument is true, and zero otherwise. A local estimate of the false discovery rate for a given value of *Z* can then be computed as
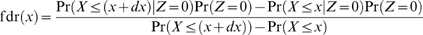
(8)


Because the empirical CDF in the Benjamini Hochberg FDR calculation is only evaluated at the data points, we evaluate Eqn. **(8)** using the continuous estimate of 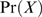 described in our formulation of the empirical Bayes procedure. The calculation is then equivalent to the local fdr, calculating the null distribution based on a Gaussian model and setting the prior null probability to 1.

### Materials and reagents

L-arginine-13C6 and L-lysine-13C615N2 were from Sigma Isotec (St. Louis, MO). The cell culture media, Roswell Park Memorial Institute-1640 (RPMI) deficient in arginine, lysine and methioine, was a custom media preparation from Caisson Laboratories (North Logan, UT). All other L-amino acids were obtained from Sigma. Dialyzed serum was obtained from SAFC-Sigma. Cell culture reagents were from Invitrogen, unless otherwise specificed. Trypsin was from Promega (Madison, WI). All other reagents and chemicals used were of the highest grade available. HeLa S3 was a kind gift from Dr. James Bradner.

### Preparation of affinity matrices

The solid-phase beads used in small-molecule immobilization and affinity chromatography is Affigel 102 (Bio-Rad) with a loading level of 12 µmol/mL suspension. Small-molecules used in this study were: k252a (Biomol, Plymouth Meeting, PA), SB-202190 (Sigma), and AP-1497 (synthesized in-house [53]). Small-molecules (1 eq.) in acetonitrile were combined with di(N-succimidyl)carbonate (3 eq.), before triethylamine (4 eq.) was added. The reaction solution was stirred at 50°C for 1 h and the activation efficiency was monitored by LC-MS. The amount of activated compound was adjusted accordingly and added to Affigel beads as needed, depletion of free activated bait molecule was monitored by LC-MS. After immobilization, vials were centrifuged, the supernatant was removed and the beads were washed with DMF (3×2 mL) and H2O (3×2 mL). The beads were subsequently suspended in 1x PBS (0.8 mL) and stored at 4°C before use. Beads loaded with 12% compound loading had approximately 18.5 nmoles of compound with the remaining bead surface underivatized and bearing the original free amine.

### SILAC labeling and affinity enrichments

The suspension cell line, HeLa S3, was grown in RPMI SILAC labeling media, prepared as previously described [54], supplemented with 2 mM L-glutamine, and 5% dialyzed fetal bovine serum (SAFC-Sigma) plus antibiotics, in a humidified atmosphere with 5% CO2 in air. Cells were grown for at least 6 cell divisions in labeling media, and expanded in spinner flasks to obtain about 35 mg protein in each state.

Separate cultures of HeLa S3 cells SILAC labeled either with L-arginine and L-lysine (light) or L-arginine-13C6 and L-lysine-13C6-15N2 (heavy) were lysed in ice-chilled ModRIPA buffer (low stringency buffer LS) containing 1% NP-40, 0.1% Na deoxycholate, 150 mM NaCl, 1 mM EDTA, 50 mM Tris, pH 7.5, and protease inhibitors (CompleteTM tablets, Roche Applied Science, Indianapolis, IN). Chilled lysates were vortexed intermittently and clarified by spinning at 14,000×g. Protein concentrations of light and heavy lysates were equalized using the Protein Assay Dye Reagent Concentrate (Biorad, Hercules CA).

In soluble competitor experiments, the appropriate amount of small-molecule (dissolved in DMSO with stocks at 110 nmoles/µL) was added to 2 mg of light HeLa S3 lysate. An equal volume of DMSO was then added to 2 mg of heavy HeLa S3 as a control. 25 µL of 50% of small-molecule-bead was added to both light and heavy pull-down tubes in soluble competitor experiments.

Affinity enrichments were incubated overnight on an end-over-end rotator at 4°C. Beads were collected by centrifugation at 1000×g, and washed twice with ModRIPA buffer before beads were combined and washed together in the third wash. Proteins enriched in SILAC affinity pull-downs were reduced and alkylated, on bead, in 2 mM DTT and 10 mM iodoacetamide respectively before adding sample buffer and heating at 70°C for 10 minutes. Proteins were resolved on a 4–12% gradient 1.5 mm thick Bis-Tris gel with MES running buffer (Nupage, Invitrogen) and Coomassie stained (Simply Blue, Invitrogen). Gel lanes were excised into 6 pieces and then further cut into 1.5 mm cubes, and proteins digested overnight with trypsin following standard protocols. Peptides from each gel slice were extracted with 0.1% TFA and cleaned up on C18 StageTips [55]. Peptides were eluted in 50 µL of 80% acetonitrile/0.1% TFA and dried down in a evaporative centrifuge to remove organic solvents. The peptides were then resuspended by vortexing in 7 µL of 0.1% TFA and analyzed by nanoflow-LC/MS with an Agilent 1100 with autosampler (HP, Palo Alto, CA) and a LTQ-Orbitrap (Thermo, Bremen Germany). Peptides were resolved on a 10 cm column, made in-house by packing a self-pulled 75 µm I.D. capillary, 15 µm tip (P-2000 laser based puller, Sutter Instruments) column with 3 µm Reprosil-C18-AQ beads (Dr. Maisch GmbH, Ammerbuch-Entringen, Germany) with an analytical flowrate of 200 nL/min and a 58 min linear gradient (∼0.57%B/min) from 0.1% formic acid in water to 0.1% formic acid/90% acetonitrile.

### MS data pre-processing

We used an MS method with a master Orbitrap full scan (60,000 resolution) and data dependent LTQ MS/MS scans for the top 5 precursors (excluding z = 1) from the Orbitrap scan. Each cycle was approximately 2 secs long. MS raw files were processed for protein identification and quantitation using extract_msn.exe (Thermo, Bremen Germany), Mascot (Ver. 2.1.03 Matrixscience, London UK), and open-source academic software, DTASupercharge and MSQuant (CEBI, http://msquant.sourceforge.net) (Figure S4). MS/MS peak lists in Mascot Generic Format were generated using extract_msn.exe and DTASupercharge (ver. 1.17, default settings) and searched with Mascot using IPI human ver.3.32 (http://ebi.ac.uk) with a concatenated decoy database containing randomized sequences from the same database [56]. Common contaminants like bovine serum albumin, trypsin etc. were also added to the database. Variable modifications used were oxidized methionine, arginine-^13^C_6_, lysine-^13^C_6_^15^N_2_, and carbamidomethyl-cysteine was a fixed modification. The precursor mass tolerance used in the search was 15 ppm and fragment mass tolerance was 0.7 Da. The Mascot result file in the appropriate format (http://msquant.sourceforge.net) was parsed by MSQuant with the following settings: bold and checked red, and parenthesized peptides were included in the list of preselected peptides, of these, peptides with score >20 were quantified. Proteins with a minimum Mascot score of 66 (at least 1 peptide with score >66) were exported to data files by MSQuant and these text files (included in Data Set S1) were used as input for our statistical analysis. Only proteins with a minimum of 2 quantifiable peptides in either replicate experiment were included in our data set. The false-positive rate for protein identification was <1% and <5% at the peptide level, as determined using the decoy database strategy.

## Supporting Information

Figure S1 Comparison of methods for estimation of the marginal probability distribution. The plots display the results of 3 different methods for inferring the marginal distribution of log2 protein ratio values from a k252a experiment at 2.5x and 5x concentrations. Using a seventh-order Poisson regression model (dotted red curve) (Efron, 2002) yielded an over-fit model of the tails of the distribution. Other similar methods proposed by (Efron, 2002) (e.g. natural splines) produced highly similar results to the Poisson regression model. Our density estimation method (green curve) yielded a more accurate model of the data. We also tested the ability to fit the data using a T distribution (dotted cyan curve), but observed that this approach in general produced overly heavy tails and therefore over-estimated the significance of data points with high (or low, where applicable) ratio values.
(0.14 MB PDF)

Figure S2 Magnitude versus amplitude plot of peptide values of a k252a experiment performed at .25x concentration. We suggest that quantitative proteomics datasets should be tested for intensity-dependent variance in ratio values in order to choose a method of summarizing values for multiple peptides identifying the same protein. Because our datasets displayed minimal intensity-dependent variance, we used the median across peptide values, but for datasets displaying intensity-dependence we suggest using an intensity-weighted average instead.
(0.12 MB PDF)

Figure S3 Robustness of inferred distributions to inference parameters. The robustness of inferred distributions to estimation parameters was demonstrated for an affinity pull-down experiment performed using k252a at .25x concentration and a miR-16 over-expression experiment. (a) The class-conditional probability distribution of log2 SILAC ratio values, Pr(X|Z = 0), was estimated using a fixed-sized window around the central peak of the data. The size of this window should be chosen such that it includes few values thought to be significant, although it has been shown that if Pr(Z = 0) >.9, the null distribution can be fit with negligible bias, even though some values in the window may not belong to the null distribution (Efron, 2004). The choice of this parameter may be based on prior knowledge regarding the expected variability of the experiment, or the percent of proteins expected to be significant. However, because the central portion of the data was well modeled by a Gaussian distribution, our results were robust over a large range of values for this parameter. (b) The marginal distribution, Pr(X), was modeled using kernel density estimation with generalized Pareto distributions fit to the tails. The percent of data used to model the generalized Pareto distributions should be chosen to reflect the region where the data becomes sparse and cannot be accurately modeled using kernel density estimation. In this work we fit the generalized Pareto distributions from −.5 to +.5 from the central peak, but have also found that estimation is extremely robust over a large range of this parameter. Although we suggest that our parameter choices are reasonable for other data sets that demonstrate similar experimental variance, an improved automated procedure may be appropriate to choose these parameters in an experiment-specific manner, for example by testing for deviations from normality to estimate the null distribution, or by testing for data sparsity to estimate the marginal distribution. In the current work, our suggested heuristics appear to perform well, with the inference results being robust to different choices for these parameters.
(0.13 MB PDF)

Figure S4 SILAC target ID data pre-processing workflow.
(0.09 MB PDF)

Figure S5 microRNA seed enrichments compared to predicted local fdrs for different parameter choices and binding site definitions. Each plot (A–F) corresponds to Figure 6a from the main text. (A–E) Seed enrichments were calculated based on 4 alternate binding site definitions, as used by (Selbach et al, 2008), as well as using predicted targets from the Target Scan database (Lewis et al, 2005), and using a bin size of 100 proteins. (F) The result from Figure 6a in the main text was reproduced using a background size of one-fifth rather than one-third.
(0.25 MB PDF)

Table S1 Small-molecule target identification. This table contains lists of target proteins inferred in each affinity pull-down experiment. The 3 first pages each present the results from 1 of the analysis methods described in our study. For each experiment (listed in the first row), we provide a list of proteins identified as targets, followed by 2 columns of binary numbers listing whether the protein is a known target or a secondary interactor, followed by a “Notes” column annotating any additional evidence suggesting that an unknown target may in fact interact with the tested small-molecule. The fourth page provides a summary of the results of each analysis method, with predictions evaluated with respect to our lists of known targets.
(0.07 MB XLS)

Table S2 microRNA statistics. This table contains a statistical description of each microRNA experiment, based on the empirical Bayes model. A description of each data column is included in the table.
(0.04 MB XLS)

References S1 List of publications referenced in the supplementary figure and table legends.
(0.07 MB PDF)

Data Set S1 MSQuant output files for small-molecule target identification experiments analyzed in this study.
(38.94 MB ZIP)
