# Supplementary figures and images for: Empirical Bayes Analysis of Quantitative Proteomics Experiments
Source: PLoS One. 2009 Oct 14;4(10):e7454. doi: 10.1371/journal.pone.0007454 (PMC2759080; doi:10.1371/journal.pone.0007454)

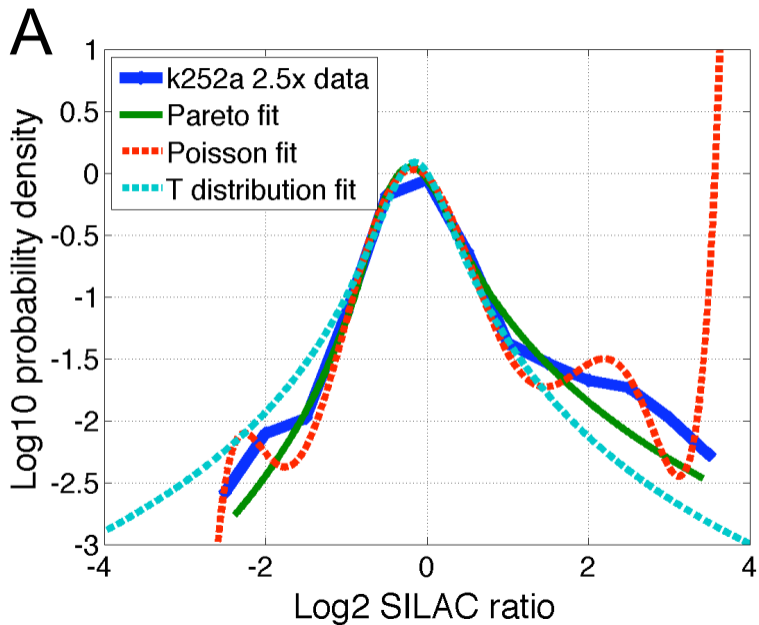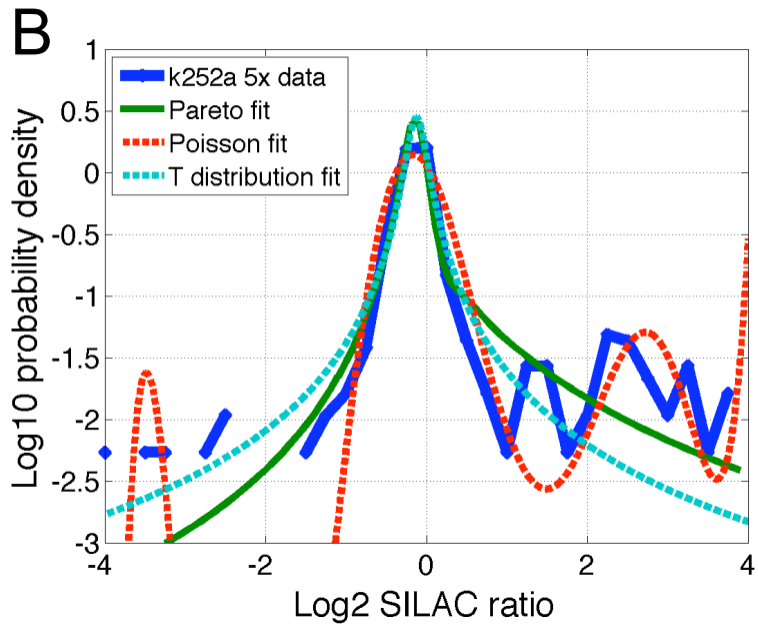

Supplement: Figure S1 — Comparison of methods for estimation of the marginal probability distribution. The plots display the results of 3 different methods for inferring the marginal distribution of log2 protein ratio values from a k252a experiment at 2.5x and 5x concentrations. Using a seventh-order Poisson regression model (dotted red curve) (Efron, 2002) yielded an over-fit model of the tails of the distribution. Other similar methods proposed by (Efron, 2002) (e.g. natural splines) produced highly similar results to the Poisson regression model. Our density estimation method (green curve) yielded a more accurate model of the data. We also tested the ability to fit the data using a T distribution (dotted cyan curve), but observed that this approach in general produced overly heavy tails and therefore over-estimated the significance of data points with high (or low, where applicable) ratio values. (0.14 MB PDF) [file pone.0007454.s001.pdf]

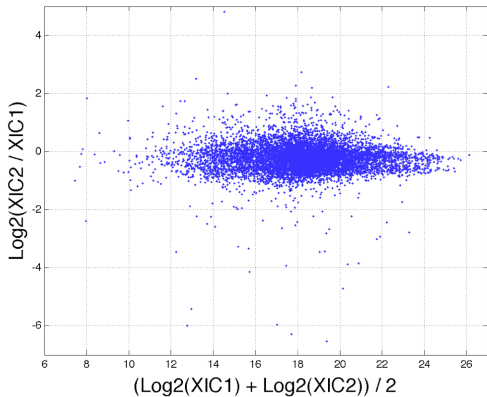

Supplement: Figure S2 — Magnitude versus amplitude plot of peptide values of a k252a experiment performed at .25x concentration. We suggest that quantitative proteomics datasets should be tested for intensity-dependent variance in ratio values in order to choose a method of summarizing values for multiple peptides identifying the same protein. Because our datasets displayed minimal intensity-dependent variance, we used the median across peptide values, but for datasets displaying intensity-dependence we suggest using an intensity-weighted average instead. (0.12 MB PDF) [file pone.0007454.s002.pdf]

# LTQ-Orbitrap data analysis workflow/protocol : SILAC for Target ID

Automated by run-pipeline.pl

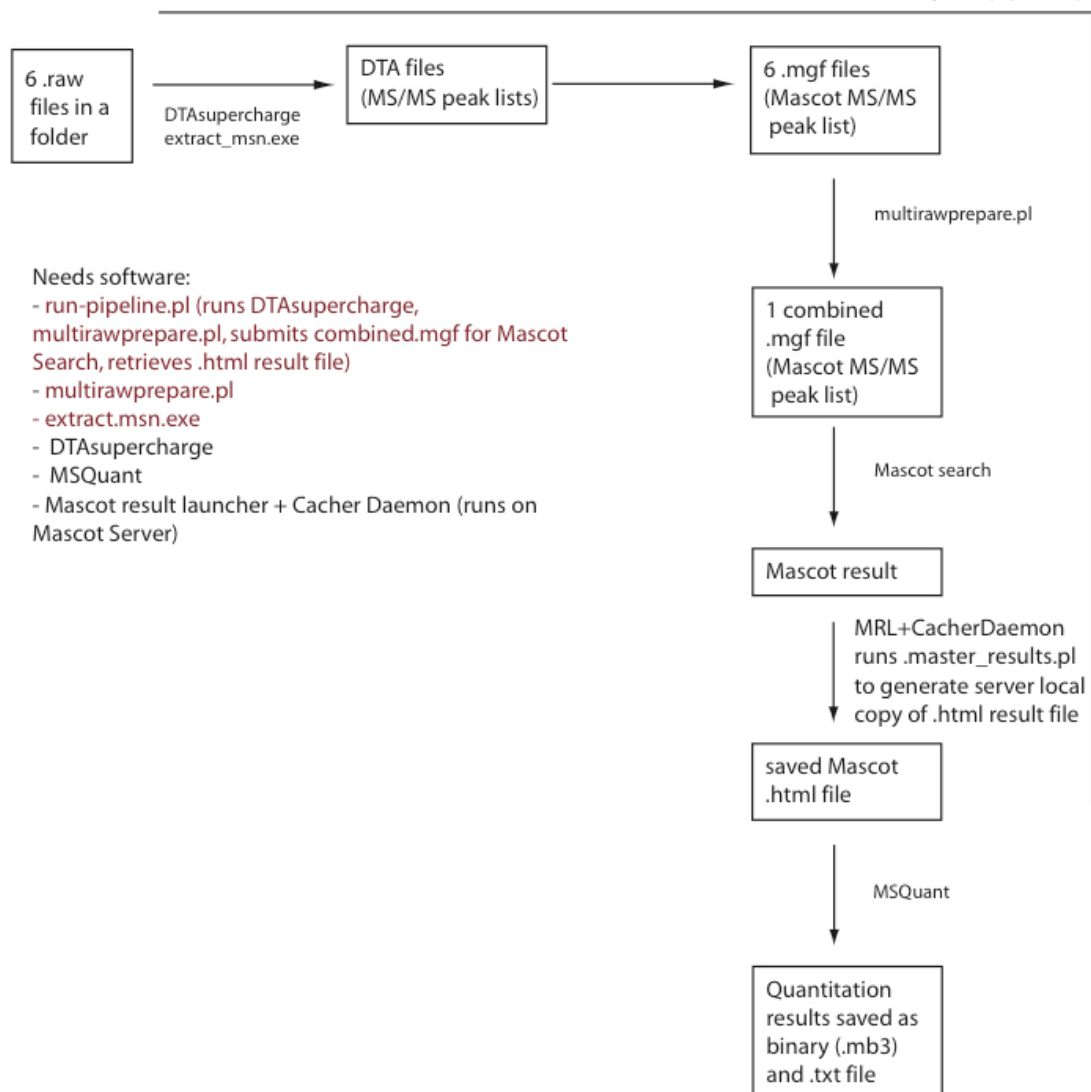

Supplement: Figure S4 — SILAC target ID data pre-processing workflow. (0.09 MB PDF) [file pone.0007454.s004.pdf]

7-mer (2-8)

A

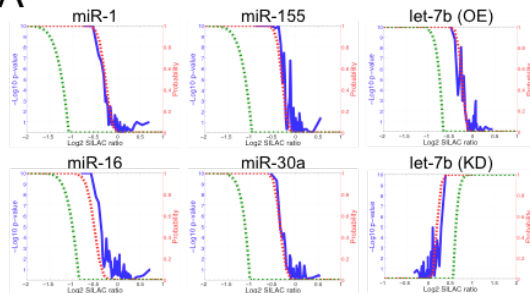

7-mer (a2-7)

B

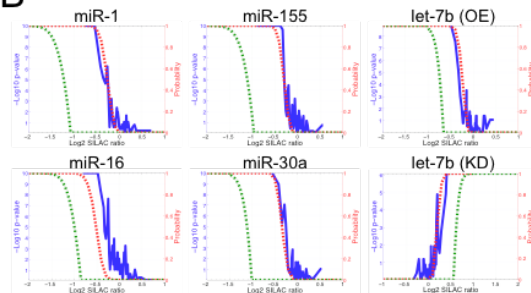

8-mer (1-8)

C

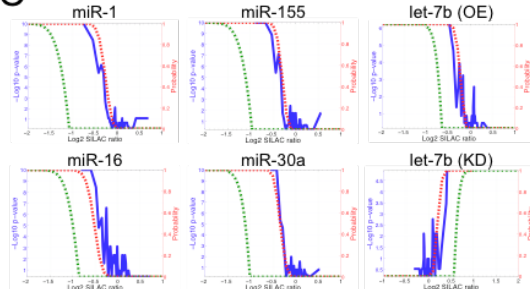

6-mer (2-7)

D

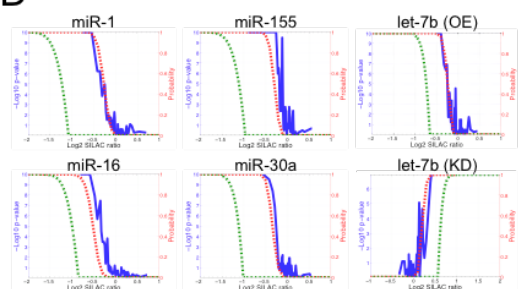

Target Scan

E

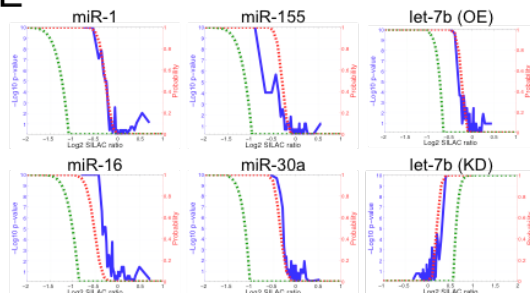7-mer (2-8), bin size 50, background size  $1/5^{\text{th}}$ 

F

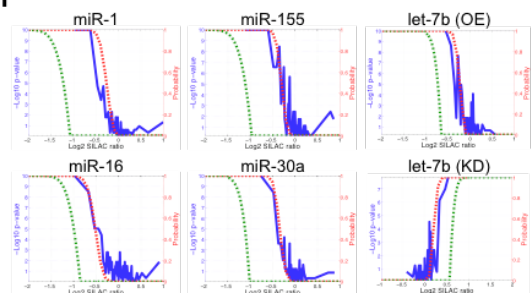

Supplement: Figure S5 — microRNA seed enrichments compared to predicted local fdrs for different parameter choices and binding site definitions. Each plot (A–F) corresponds to Figure 6a from the main text. (A–E) Seed enrichments were calculated based on 4 alternate binding site definitions, as used by (Selbach et al, 2008), as well as using predicted targets from the Target Scan database (Lewis et al, 2005), and using a bin size of 100 proteins. (F) The result from Figure 6a in the main text was reproduced using a background size of one-fifth rather than one-third. (0.25 MB PDF) [file pone.0007454.s005.pdf]
