# Supplementary material for: Empirical Bayes Analysis of Quantitative Proteomics Experiments
Source: PLoS One. 2009 Oct 14;4(10):e7454. doi: 10.1371/journal.pone.0007454 (PMC2759080; doi:10.1371/journal.pone.0007454)
Supplement: Figure S3 — Robustness of inferred distributions to inference parameters. The robustness of inferred distributions to estimation parameters was demonstrated for an affinity pull-down experiment performed using k252a at .25x concentration and a miR-16 over-expression experiment. (a) The class-conditional probability distribution of log2 SILAC ratio values, Pr(X|Z = 0), was estimated using a fixed-sized window around the central peak of the data. The size of this window should be chosen such that it includes few values thought to be significant, although it has been shown that if Pr(Z = 0) >.9, the null distribution can be fit with negligible bias, even though some values in the window may not belong to the null distribution (Efron, 2004). The choice of this parameter may be based on prior knowledge regarding the expected variability of the experiment, or the percent of proteins expected to be significant. However, because the central portion of the data was well modeled by a Gaussian distribution, our results were robust over a large range of values for this parameter. (b) The marginal distribution, Pr(X), was modeled using kernel density estimation with generalized Pareto distributions fit to the tails. The percent of data used to model the generalized Pareto distributions should be chosen to reflect the region where the data becomes sparse and cannot be accurately modeled using kernel density estimation. In this work we fit the generalized Pareto distributions from −.5 to +.5 from the central peak, but have also found that estimation is extremely robust over a large range of this parameter. Although we suggest that our parameter choices are reasonable for other data sets that demonstrate similar experimental variance, an improved automated procedure may be appropriate to choose these parameters in an experiment-specific manner, for example by testing for deviations from normality to estimate the null distribution, or by testing for data sparsity to estimate the marginal distri [file pone.0007454.s003.pdf]

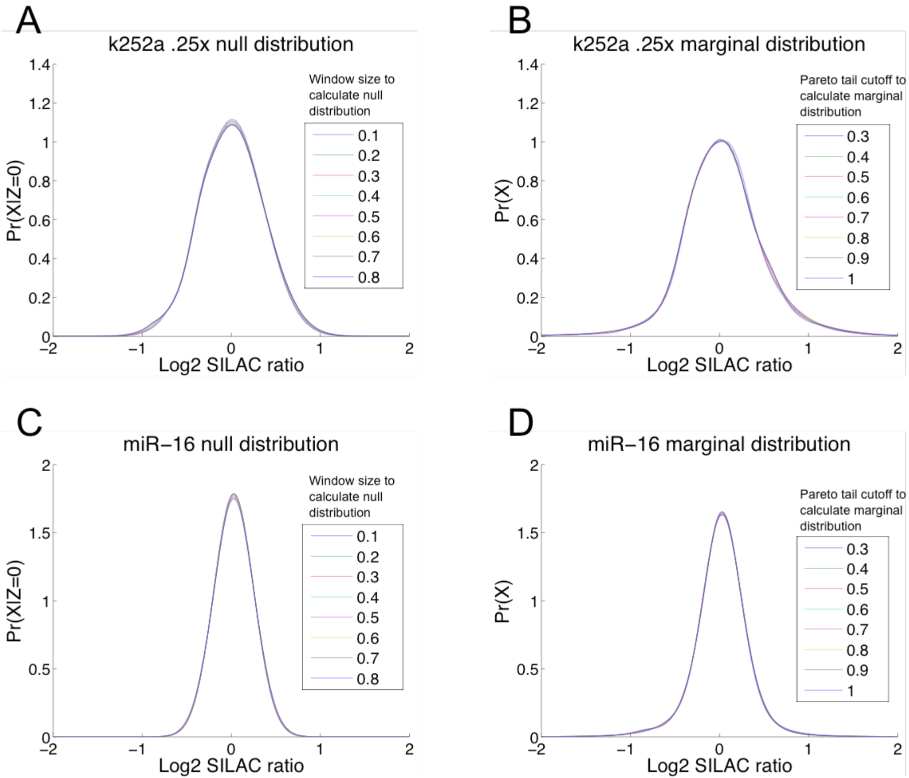
A
k252a .25x null distribution
Window size to calculate null distribution
0.1
0.2
0.3
0.4
0.5
0.6
0.7
0.8
Pr(X|Z=0)
Log2 SILAC ratio
B
k252a .25x marginal distribution
Pareto tail cutoff to calculate marginal distribution
0.3
0.4
0.5
0.6
0.7
0.8
0.9
1
Pr(X)
Log2 SILAC ratio
C
miR–16 null distribution
Window size to calculate null distribution
Pr(X|Z=0)
Log2 SILAC ratio
D
miR–16 marginal distribution
Pareto tail cutoff to calculate marginal distribution
Pr(X)
Log2 SILAC ratio
