# Supplementary material for: Empirical Bayes Analysis of Quantitative Proteomics Experiments
Source: PLoS One. 2009 Oct 14;4(10):e7454. doi: 10.1371/journal.pone.0007454 (PMC2759080; doi:10.1371/journal.pone.0007454)
Supplement: References S1 — List of publications referenced in the supplementary figure and table legends. (0.07 MB PDF) [file pone.0007454.s008.pdf]

REFERENCES

Dennis G, Jr., Sherman BT, Hosack DA, Yang J, Gao W, Lane HC, Lempicki RA (2003) DAVID: Database for Annotation, Visualization, and Integrated Discovery. *Genome biology* **4:** P3.

Efron B (2002) Empirical Bayes methods and false discovery rates for microarrays. *Genetic Epidemiology* **23:** 70-86.

Efron B (2004) Large-Scale Simultaneous Hypothesis Testing: The Choice of a Null Hypothesis. *Journal of the American Statistical Association* **99:** 96-104.

Lewis BP, Burge CB, Bartel DP (2005) Conserved seed pairing, often flanked by adenosines, indicates that thousands of human genes are microRNA targets. *Cell* **120:** 15-20.

Schulze WX, Mann M (2004) A novel proteomic screen for peptide-protein interactions. *J Biol Chem* **279:** 10756-10764.

Selbach M, Schwanhausser B, Thierfelder N, Fang Z, Khanin R, Rajewsky N (2008) Widespread changes in protein synthesis induced by microRNAs. *Nature* **455:** 58-63.
